# Development and Evaluation of an Immuno-MALDI-TOF Mass Spectrometry Approach for Quantification of the Abrin Toxin in Complex Food Matrices

**DOI:** 10.3390/toxins13010052

**Published:** 2021-01-13

**Authors:** Sandrine Livet, Sylvia Worbs, Hervé Volland, Stéphanie Simon, Martin B. Dorner, François Fenaille, Brigitte G. Dorner, François Becher

**Affiliations:** 1 CEA, INRAE, Département Médicaments et Technologies pour la Santé (DMTS), Université Paris Saclay, SPI, 91191 Gif-sur-Yvette, France; sandrine.livet@gmail.com (S.L.); herve.volland@cea.fr (H.V.); stephanie.simon@cea.fr (S.S.); francois.fenaille@cea.fr (F.F.); 2 Biological Toxins, Centre for Biological Threats and Special Pathogens, Robert Koch Institute, 13353 Berlin, Germany; worbss@rki.de (S.W.); dornerm@rki.de (M.B.D.); dornerb@rki.de (B.G.D.)

**Keywords:** abrin, MALDI-TOF, mass spectrometry, immunoaffinity, quantification, food matrices

## Abstract

The toxin abrin found in the seeds of *Abrus precatorius* has attracted much attention regarding criminal and terroristic misuse over the past decade. Progress in analytical methods for a rapid and unambiguous identification of low abrin concentrations in complex matrices is essential. Here, we report on the development and evaluation of a MALDI-TOF mass spectrometry approach for the fast, sensitive and robust abrin isolectin identification, differentiation and quantification in complex food matrices. The method combines immunoaffinity-enrichment with specific abrin antibodies, accelerated trypsin digestion and the subsequent MALDI-TOF analysis of abrin peptides using labeled peptides for quantification purposes. Following the optimization of the workflow, common and isoform-specific peptides were detected resulting in a ~38% sequence coverage of abrin when testing ng-amounts of the toxin. The lower limit of detection was established at 40 ng/mL in milk and apple juice. Isotope-labeled versions of abundant peptides with high ionization efficiency were added. The quantitative evaluation demonstrated an assay variability at or below 22% with a linear range up to 800 ng/mL. MALDI-TOF mass spectrometry allows for a simple and fast (<5 min) analysis of abrin peptides, without a time-consuming peptide chromatographic separation, thus constituting a relevant alternative to liquid chromatography-tandem mass spectrometry.

## 1. Introduction

Abrin is a protein toxin contained in the seeds of the plant *Abrus precatorius*, found in tropical regions [1]. Abrin belongs to the ribosomal inactivating protein class II (RIP II), similarly to the related toxin ricin from *Ricinus communis* [2,3]. Abrin and ricin toxins are both composed of A and B-polypeptide chains, which are approximately 30–32 kDa each, resulting in a molecular weight of approximately 60–64 kDa with several N-glycosylation sites. Both toxins occur in many isoforms in plants, e.g., abrin-a, abrin-b, abrin-c and abrin-d (sequence similarity ≈78%) or ricin D and ricin E (sequence similarity ≈97%), respectively [4,5]. Because of their high toxicity in humans, both toxins are considered as relevant agents for potential criminal and terroristic misuse [6]. In such a scenario, a reliable and fast identification of toxins in potentially contaminated environmental samples at low level is of great importance. The identification of toxins is based either on antigen recognition by antibodies, e.g., ELISA, and on-site detection methods such as lateral flow assays, or on sequence characterization by mass spectrometry. Pure antibody-based methods are most sensitive but may generate false-positive or false-negative results [7]. On the other hand, mass spectrometry (MS)-based methods, optionally combined with enrichment by antibodies for improved detection sensitivity and matrix compatibility, provide a precise mass measurement of protein sequences for specific toxin identification, differentiation from proteins with high sequence homology and robust quantification. Mass spectrometry-based proteomic identification of toxins involves digestion into peptides by trypsin, eventually detected by MS either using electrospray ionization (ESI) or matrix-assisted laser desorption/ionization (MALDI) [8]. Liquid chromatography coupled with ESI and tandem mass spectrometry (LC-ESI-MS/MS) was mostly reported for ricin, enabling the identification in several environmental or food matrices at low ng/mL concentrations [8,9,10,11,12,13,14]. Along this line, abrin detection and quantification by tandem mass spectrometry at high resolution (LC-ESI-MS/HRMS) was recently reported by our group [5]. As an alternative, MALDI-time-of-flight (TOF) MS has some advantages over ESI-MS, notably the speed of analysis and the simplicity, with the absence of a prior chromatographic separation. Nevertheless, only few MALDI-TOF MS assays have been described for ricin [12,15,16,17], and none for abrin. 

In this report, we describe a fast, sensitive and robust method for abrin identification and quantification by combining immunoaffinity-enrichment and MALDI-TOF MS analysis (iMALDI) with isotope dilution [18,19]. The assay enabled the detection of abrin in the low ng/mL range in milk and apple juice, and provided a quantitative performance similar to that of LC-ESI-MS/HRMS methods. 

## 2. Results

The objective of this work was to widen the scope of the application of the LC-ESI-MS/HRMS assay previously reported by our group for abrin identification and quantification in complex matrices [5], taking advantage of MALDI-TOF MS’s simplicity, time efficiency and broader availability. The MS detection of toxins in different matrices at high sensitivity requires selective sample preparation strategies to reduce ionization suppression. Sample preparation is even more crucial with MALDI-TOF MS, because this technique is most frequently not combined with prior liquid chromatography separation. In this context, the enrichment by antibodies targeting the protein in a specific manner was considered as most appropriate, and was implemented first, before further assay evaluations.

### 2.1. Assay Development

We reported previously an immuno-enrichment protocol based on the immobilization of four different monoclonal antibodies (mAbs) directed against abrin [5], namely antibodies AP430, AP3659, AP3808 and AP476 [Worbs et al., manuscript in preparation]. Rapid on-beads trypsin digestion combined with LC-ESI-MS/HRMS was found to be efficient for the release of unique peptides of the toxin following enrichment [13,20]. When applying the on-beads protocol to the MALDI-TOF MS detection of abrin spiked at 250 ng/mL in a simple bovine serum albumin (BSA) buffer, a low or no signal was observed for abrin peptides in the MS spectrum (Figure S1). We assumed that the ionization of abrin peptides was suppressed by peptides released by the trypsin from BSA (i.e., the albumin buffer or coated on beads), from abrin antibodies or by polymers (i.e., from magnetic beads conditioning medium or washing buffers). 

**Figure 1:**
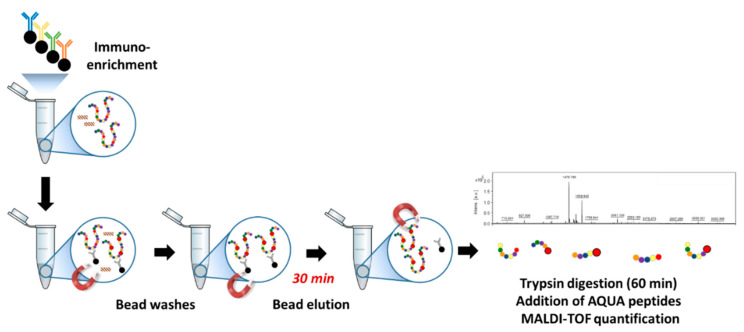
Workflow for abrin detection by immuno-MALDI-TOF mass spectrometry (MS). The workflow includes immunoenrichment with four different abrin-specific mAbs, several beads washes, acid-elution of abrin from the beads before digestion by trypsin and MALDI-TOF quantification of abrin specific peptides.

**Figure 2:**
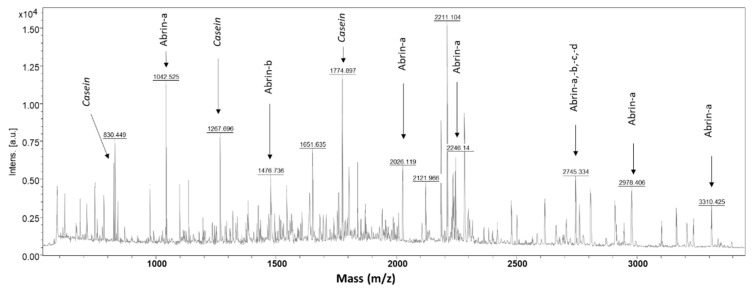
MALDI-TOF spectra of abrin at 250 ng/mL in milk 20% in final optimized assay conditions. The most intense abrin peptides and isoform specificity are indicated. The residual signal from casein peptides is shown.

The protocol optimization included the evaluation of additional beads washes [21] for getting rid of polymeric contaminants and the determination of the best conditions for the elution of abrin from the beads before the digestion of eluted abrin by trypsin (Figure S2). The best signal of abrin peptides was obtained using a low percentage of organic solvent, i.e., 10% acetonitrile, in acid elution conditions (Figure S2). The final protocol for abrin determination by immuno-MALDI-TOF MS is illustrated in Figure 1, including abrin elution from the beads and rapid digestion by trypsin within 60 min [5] of eluted abrin resulting in a total assay time of ≈2.5 h. Under these conditions, abrin peptides dominated the MS spectrum even when the capture was performed from milk samples spiked with abrin at 250 ng/mL (Figure 2). 

### 2.2. Evaluation of Immuno-MALDI-TOF MS for the Detection and Quantification of Abrin in Food Matrices

The sequence coverage in the final assay conditions was determined at a mid-concentration range of 250 ng/mL in buffer. Based on the amino-acid sequence of abrin-a, the sequence coverage was determined at ~38%, including five peptides shared by the four isoforms and eight unique peptides to abrin-a (Table 1, Figure S3). In addition, 3 peptides unique to abrin-b and two unique to abrin-c,-d were detected (Table 1). As expected, arginine-containing peptides dominated in the mass spectrum (23 peptides) over those that contained lysine (two peptides) due to their superior detection/ionization efficiency [22]. Two long peptides previously undetected by LC-ESI-MS/HRMS operated in data-dependent acquisition [5], i.e., VSIQTGTAFQPDAAMISLENNWDNLSR and SALVLSAESSSMGGTLTVQTNEYLMR, were well detected and identified by MALDI-TOF MS, thus providing a complementary sequence coverage. Interestingly, the peptide SALVLSAESSSMGGTLTVQTNEYLMR is common to the four abrin isoforms. 

**Figure 3:**
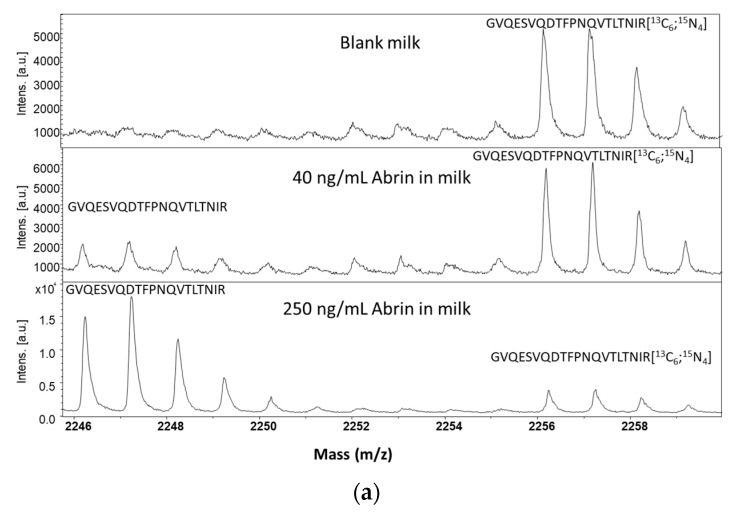

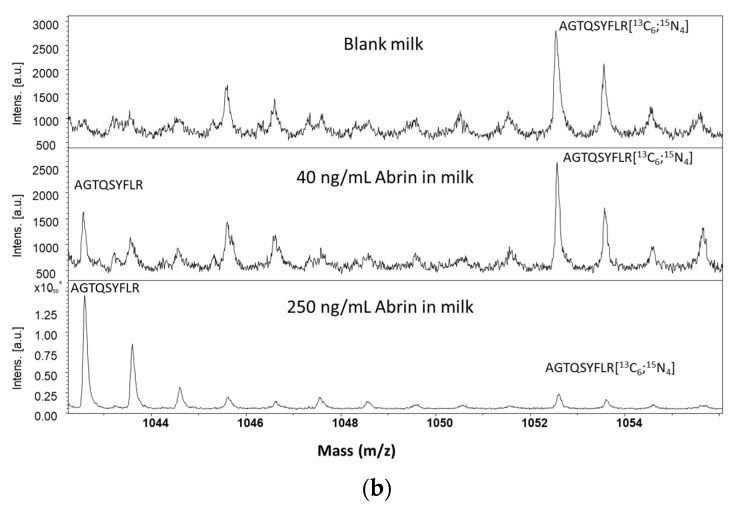
Determination of the limit of detection (LOD) for abrin in 20% milk by immuno-MALDI-TOF MS. MS Signal of peptides (**a**) GVQESVQDTFPNQVTLTNIR and (**b**) AGTQSYFLR in a blank sample (top), at 40 ng/mL abrin (middle) and at 250 ng/mL abrin (bottom). The signal of the labeled peptides is indicated on the MS spectra.

**Figure 4:**
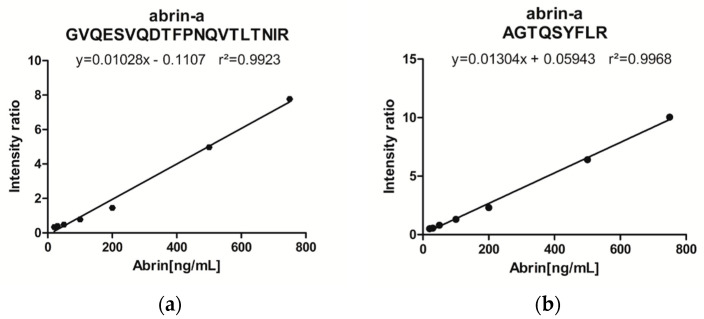
Linearity of abrin quantification in 20% milk by immuno-MALDI-TOF MS. Calibration curves were obtained with the peptides selected for abrin quantification, peptides (**a**) GVQESVQDTFPNQVTLTNIR and (**b**) AGTQSYFLR. The intensity ratio with the labeled peptide to the spiked abrin concentration is reported.

Peptides were selected for the quantitative assay based on the usual recommendations [23] among the most intense peaks in the MS spectrum. The peptides observed in the MALDI-TOF spectrum with pyroglutamate formation from N-terminal glutamine, oxidation or miscleavage were excluded, as well as all peptides containing amino-acids prone to chemical modification like methionine or cysteine residues (Table 1). The peptides AGTQSYFLR and GVQESVQDTFPNQVTLTNIR of abrin-a were selected for the quantitative experiments. Additionally, the peptide YLFTGTQQYSLR, representative of abrin-b, was included. Because abrin-a accounts for ~75% of the total abrin content in the lactosyl-purified material from *Abrus precatorius* seeds (whereas abrin-b amounts to ~15%) [5], the quantitative value of the protocol was assessed with the two abrin-a peptides. For the same reason, peptides from the minor isoforms abrin-c or abrin-d were not included in the quantitative evaluation. To ensure assay specificity and improve the quantitative accuracy, the selected peptides were obtained in labeled form with [^13^C_6_;^15^N_4_]-arginine. The limit of detection (LOD) of the immuno-MALDI-TOF MS detection was determined by spiking increasing concentrations of purified abrin in 20% milk. LOD was determined as the lowest spiked concentration, resulting in a signal to noise ratio around 3 for the 2 abrin-a peptides. In this condition, LOD was found at 40 ng/mL in a 20% milk sample, as illustrated in Figure 3, with the signal of the peptides AGTQSYFLR and GVQESVQDTFPNQVTLTNIR, which corresponds to approximately 400 pg/6.5 fmol abrin, deposited on each spot of the MALDI plate. At the LOD level, four additional abrin peptides were also detected, including the long peptide SALVLSAESSSMGGTLTVQTNEYLMR common to the four isoforms. Linearity was determined up to 800 ng/mL resulting in a linear range of around 50-fold (Figure 4). Most importantly, our method demonstrated adequate sensitivity for the detection of abrin in food samples, considering the estimated lethal doses of abrin after oral administration at approximately 5 to 20 mg/kg of body weight [24]. Regarding quantification precision, intra-assay variability was evaluated in milk, as a representative protein-rich matrix, and apple juice, by spiking low and medium concentrations at 75 and 250 ng/mL, respectively, in five independent replicates each. Assay precisions (CV%) were determined, ranging between 6.9 and 22% in milk or between 8.4 and 16.5% in apple juice, for the peptides AGTQSYFLR and GVQESVQDTFPNQVTLTNIR (Table 2), respectively. Taking all these results together, the protocol proved efficient for the detection and quantification of abrin in milk and apple juice (Table 2). A further evaluation could be done in a diversity of food matrices, including solid and other sugar-rich matrices where abrin enrichment could be impacted.

## 3. Discussion

We previously reported a quantitative mass spectrometry-based assay for abrin in complex matrices. After the immuno-affinity enrichment of the toxin and on-beads trypsin digestion, both shared and isoform-specific peptides were monitored by multiplex LC-ESI-MS/HRMS on a quadrupole-Orbitrap high-resolution mass spectrometer [5]. With the objective of widening the scope of the application of our assay, we evaluated here the relevance of detecting and quantifying abrin peptides by immuno-MALDI-TOF MS as an alternative to LC-ESI-MS/HRMS. When the on-beads digestion protocol [5,13,20] was first tested with MALDI-TOF MS, we observed high background interferences in the MS spectrum and signal suppression due to peptides released by proteins from the matrix and/or proteins coated on the beads. Additional washes and the elution of abrin from the beads were necessary. In the final conditions, linear calibration curves were obtained down to 40 ng/mL. These modifications to the workflow were key for the successful implementation of the MALDI-TOF MS detection of abrin. 

This work is, to our knowledge, the first development and evaluation of an immuno-MALDI-TOF MS approach for abrin identification in complex matrices. In the final assay conditions, the LOD was determined at ~40 ng/mL in milk or apple juice. The sensitivity of the new protocol is in line with previous immuno-MALDI-TOF MS methods reported for ricin identification [16,17,25], considering that ricin has minimum lethal doses similar to abrin [6,24]. Of note, a time consuming ZipTip separation was necessary in those ricin assays, to purify and concentrate ricin before the MALDI-TOF MS analysis. In our protocol, abrin eluted from the beads was directly spotted onto the MALDI plate, greatly simplifying and shortening the workflow. In addition, the quantitative value of MALDI-TOF MS was not evaluated for abrin or ricin toxins in previous studies, regarding CV% or linearity in complex matrices. Our protocol was found to be quantitative, with an intra-day variability between 6.9% and 22% in milk or apple juice and a linear response between 40 and 800 ng/mL in both matrices. Interestingly, we found roughly similar quantitative performance of immuno-MALDI-TOF MS compared to the LC-ESI-MS/HRMS assay previously reported, which showed a variability between 2.6% and 17.3% [5]. The sensitivity of LC-ESI-MS/HRMS was superior by a factor of five, inherent to the higher sample volume injected onto the LC column, i.e., 20 µL, in comparison to only 1 µL as usually deposited on each MALDI spot. Therefore, the detection sensitivity of the MALDI method can be further improved by minimizing as much as possible the sample volume before analysis.

We have shown here that the careful selection of quantitative peptides, the addition of the labeled version of those peptides and the optimization of the sample preparation allowed for a precise quantitative determination of abrin by MALDI-TOF MS. The simplification and speed-up of the assay procedure makes the immuno-MALDI-TOF MS procedure an attractive alternative for expert laboratories when performing real sample analyses. Moreover, the immuno-MALDI-TOF MS approach delivers additional peptides not detectable by LC-ESI-MS/HRMS [5], thus complementing the sequence coverage. 

## 4. Conclusions

The new method is intended to be implemented in regulatory laboratories where LC-ESI-MS/MS instruments are not available and also for fast risk assessment, providing a first response in advance to the LC-ESI-MS/HRMS measurements. To this aim, we showed that MALDI-TOF MS can constitute an efficient alternative to LC-ESI-MS/HRMS. More research would be needed to better evaluate the specificity and LOD in the diverse matrices that may be investigated in a biodefense scenario. The assay could also be combined with efficient enzymatic assays where substrates depurinated by abrin or ricin after incubation might be detected by MALDI-TOF MS [9,21,26].

## 5. Materials and Methods 

### 5.1. Safety Precaution

Due to its high toxicity, experiments using abrin were performed in a biosafety level-2 cabinet equipped with a HEPA filter. Only trained personnel were allowed to handle the toxin while wearing personal protection equipment and following specified safety protocols. Abrin-contaminated solutions and consumables were inactivated overnight using 2 M NaOH.

### 5.2. Chemicals and Materials

*Abrus* seeds were purchased from B & T World Seeds (Aigues-Vives, France). Abrin was purified from *Abrus precatorius* seeds as described in [5]. Sequencing-grade modified trypsin was obtained from Promega Corporation (Charbonnières-les-Bains, France). RapiGest SF Surfactant was purchased from Waters Corporation (Milford, MA, USA). Dynabeads M-280 tosylactivated magnetic beads were obtained from Invitrogen (Life Technologies, Oslo, Norway). Labeled peptides for quantification were synthesized in Pepotec grade by ThermoFisher Scientific (Paisley, UK). Water (ChromaSolve LC-MS), acetonitrile (ACN, HPLC-grade) and formic acid were obtained from Honeywell/Riedel-de Haen (Seelze, Germany) and VWR chemicals (Fontenay sous Bois, France), respectively. Trifluoroacetic acid (TFA) and all other chemicals were purchased from Sigma-Aldrich (Saint Quentin Fallavier, France) or VWR Chemicals (Fontenay sous Bois, France). For all reactions, LoBind Eppendorf tubes (Dutscher, Brumath, France) were used.

### 5.3. Preparation of mAb-Coated Beads

The four different monoclonal antibodies used for abrin capture (namely AP430, AP3659, AP3808 and AP476) were produced in-house at RKI using a formaldehyde inactivated mixture of abrin and agglutinin for immunization of mice [Worbs et al., manuscript in preparation]. On-beads immobilization was done according to the manufacturer’s instruction using 120 μg of antibody coupled to 12.6 mg of tosylactivated Dynabeads. 

### 5.4. Abrin Extraction and Matrix-Assisted Laser Desorption/Ionization Time-of-Flight Analysis

A 500 μL portion of toxin-containing samples, diluted in PBS containing 0.05% Tween, was incubated with 0.32 mg of antibody-coated magnetic beads (corresponding to 3 μg of antibody per reaction) for 1 h in a deep well plate on row1. The deep well plate was then placed into a KingFisher Duo Prime magnetic particle processor (Thermo Fisher Scientific, Waltham, MA, USA) for automated bead washing, which included two washes with 1 mL each of PBS containing 0.05% Tween followed by two washes with 0.5 mL of water [21]. The beads were eluted from the KingFisher Duo prime into 250 μL of water. The beads were transferred to an Eppendorf tube, the tubes were placed on a magnet, and the supernatant was removed. The beads were resuspended in 10 μL of 10% acetonitrile, and 0.1% TFA during 30 min for the elution of abrin. RapiGest SF (0.05% in 500 mM ammonium bicarbonate) was added before the supernatant was transferred to a new Eppendorf tube. The tubes were heated at 95 °C for 15 min to induce denaturation. After cooling to room temperature, 0.5 µL of sequencing-grade-modified trypsin at 100 µg/µL was used for digestion at 37 °C in a bath-type sonicator (Advantage Lab, Darmstadt, Germany) for 1 h. The digestion was stopped by adding 5 µL of 1 M HCl and incubated at 37 °C for 45 min, before centrifugation. A 5-μL aliquot was mixed with 5 μL of matrix solution consisting of α-cyano-4-hydroxycinnamic acid at 5 mg/mL in 50% acetonitrile, 0.1% TFA [27] and 1.5 µL of labeled peptides AGTQSYFLR [^13^C_6_;^15^N_4_] and GVQESVQDTFPNQVTLTNIR [^13^C_6_;^15^N_4_] at 600 nM and 400 nM, respectively. A 1-μL aliquot of this mixture was spotted on a 96-spot MBT Biotarget 96 plate (Bruker, Wissembourg, France). Analyses were performed using an UltrafleXtreme instrument (Bruker Daltonics, Bremen, Germany) operating in the reflectron positive ion mode. MS spectra were acquired at a 2 kHz laser repetition rate in the positive reflector ion mode, with a 20 kV acceleration voltage and an extraction delay of 130 ns. The spectra were obtained by accumulating 5000 shots over the 500–5000 *m*/*z* range.

## Figures and Tables

**Table 1:** Abrin peptides identified by immuno-MALDI-TOF at 250 ng/mL in buffer. Masses (*m*/*z*) were determined after the internal calibration of the mass spectrum using trypsin peptides at *m*/*z* 842.5094, 1774.8975, 2211.1040 and 2283.1802 Da. Amino-acid residues surrounding each peptide are indicated in brackets. Matching proteins: a is abrin-a (uniprot P11140), b is abrin-b (uniprot Q06077), c is abrin-c (uniprot P28590), d is abrin-d (uniprot Q06076), agg is *Abrus precatorius* agglutinin (uniprot Q9M6E9).

Peptides	*m*/*z* [M + H]^+^ Theo	*m*/*z* [M + H]^+^ Obs	Difference (ppm)	Matching Protein	Modifications
(R) YEPTVR (I)	764.3937	764.39	−1	a,b,c,d	
(R) WAHQSR (Q)	784.3849	784.38	−1	a, agg	
(K) QFIEALR (E)	859.4672	859.47	−1	a,b,c,d	Pyroglutamate
(K) QFIEALR (E)	876.4938	876.49	−4	a,b,c,d	
**(R) AGTQSYFLR (D)**	**1042.5316**	**1042.53**	**−2**	**a**	
(K) QFIEALRER (L)	1144.6109	1144.60	−10	a	Pyroglutamate; Miscleavage
(R) QQIPLGLQALR (H)	1236.7423	1236.75	2	b	
**(R) YLFTGTQQYSLR (F)**	**1476.7482**	**1476.75**	**1**	**b**	
(K) DRLEENQLWTLK (S)	1544.8067	1544.81	5	a,b,c,d	Miscleavage
(K) EQQWALYTDGSIR (S)	1566.7547	1566.76	0	a,b,c,d	
(R) DAPASASTYLFPGTQR (Y)	1681.8180	1681.82	1	c,d	
(K) QGSPIVLMACSNGWASQR (W)	1887.8840	1887.93	23	b,c,d	Pyroglutamate
(R) NDGSIYNLHDDMVMDVK (R)	1965.8681	1965.89	10	b,c	
(R) QQIPLGLQALTHGISFFR (S)	2009.0967	2009.10	−1	a	Pyroglutamate
**(R) QQIPLGLQALTHGISFFR (S)**	**2026.1233**	**2026.12**	−1	**a**	
(R) GGLIHDIPVLPDPTTLQER (N)	2071.1182	2071.12	−1	a	
(R) NDGSIYNLHDDMVMDVKR (S)	2121.9692	2121.96	−3	b,c	Miscleavage
(R) LTGGLIHGIPVLPDPTTLQER (N)	2227.2445	2227.24	−2	b	
**(R) GVQESVQDTFPNQVTLTNIR (N)**	**2246.1412**	**2246.14**	**−1**	**a**	
(R) LTGGLIHDIPVLPDPTTVEER (N)	2272.2183	2272.23	3	c,d	
(K) EIILHPYHGKPNQIWLTLF (-)	2319.2648	2319.29	9	b,c	
(R) LRGGLIHDIPVLPDPTTLQER (N)	2340.3034	2340.31	2	a	Miscleavage
**(K) SALVLSAESSSMGGTLTVQTNEYLMR (Q)**	**2745.3434**	**2745.35**	**1**	**a,b,c,d**	
(K) SALVLSAESSSMGGTLTVQTNEYLMR (Q)	2761.3383	2761.34	1	a,b,c,d	Oxidation
**(R) VSIQTGTAFQPDAAMISLENNWDNLSR (G)**	**2978.4313**	**2978.44**	**3**	**a**	
**(R) DAPSSASDYLFTGTDQHSLPFYGTYGDLER (W)**	**3310.4811**	**3310.50**	**5**	**a**	

In bold: most intense peptides in the MS spectrum.

**Table 2:** Evaluation of the immuno-MALDI-TOF assay performances for abrin measurement in food matrices.

		Peptides	
Matrix		GVQESVQDTFPNQVTLTNIR	AGTQSYFLR
**Milk**	LOD (ng/mL)	40.0	40.0
	CV% 75 ng/mL	13.0	6.9
	CV% 250 ng/mL	17.3	22.0
**Apple juice**	LOD (ng/mL)	40.0	40.0
	CV% 75 ng/mL	8.4	16.5
	CV% 250 ng/mL	15.5	11.0

## Data Availability

Data sharing not applicable.

## Supplementary Materials

The following are available online at https://www.mdpi.com/2072-6651/13/1/52/s1, Figure S1: MALDI-TOF spectra of abrin at 250 ng/mL in buffer after immunocapture with on-beads digestion conditions, Figure S2: Optimization of abrin elution conditions, Figure S3: Sequence coverage of abrin at 250 ng/mL by MALDI-TOF.
