# Supplementary material for: Development and Evaluation of an Immuno-MALDI-TOF Mass Spectrometry Approach for Quantification of the Abrin Toxin in Complex Food Matrices
Source: Toxins (Basel). 2021 Jan 13;13(1):52. doi: 10.3390/toxins13010052 (PMC7828309; doi:10.3390/toxins13010052)
Supplement: Supplementary file 1 [file toxins-13-00052-s001.pdf]

Supplementary Materials: Development and Evaluation of an Immuno-MALDI-TOF Mass Spectrometry Approach for Quantification of the Abrin Toxin in Complex Food Matrices

Sandrine Livet, Sylvia Worbs, Hervé Volland, Stéphanie Simon, Martin B Dorner, François Fenaille, Brigitte G Dorner and François Becher

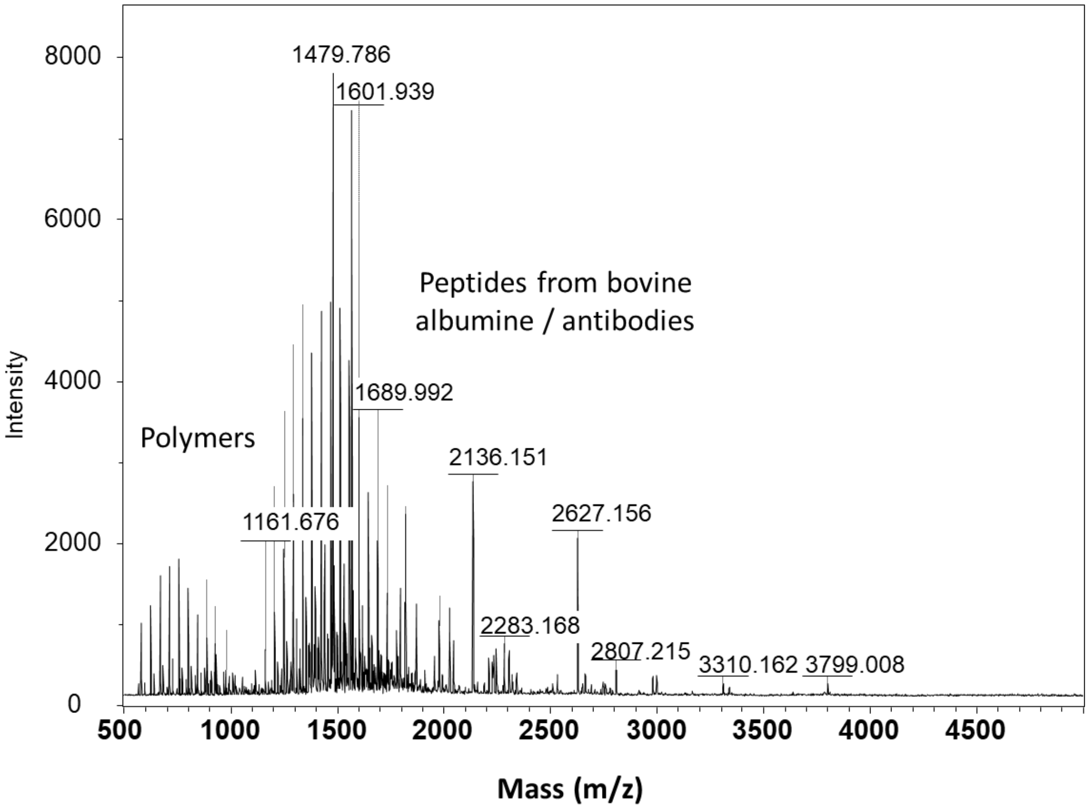

Figure S1. MALDI-TOF spectra of abrin at 250 ng/mL in buffer after immunocapture with on-beads digestion conditions.

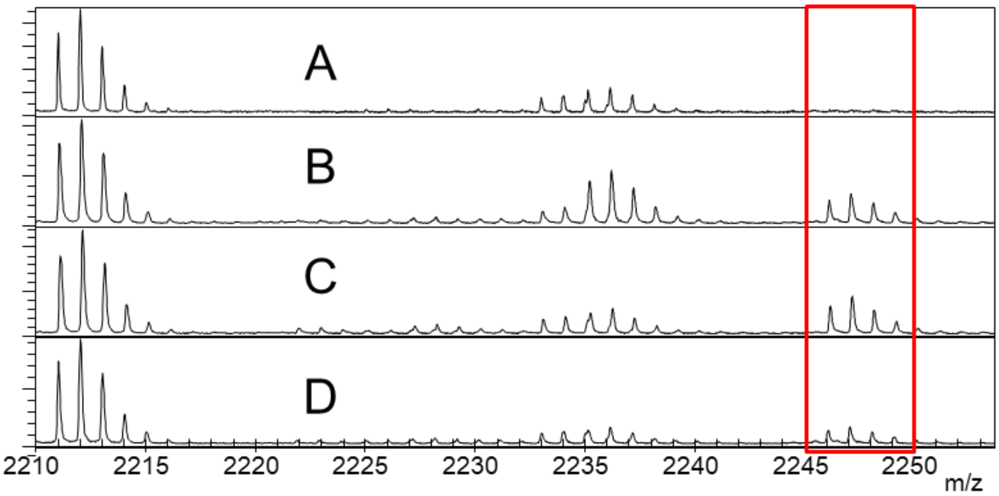

Figure S2. Optimization of abrin elution conditions. The red box indicates the signal of peptide GVQESVQDTFPNQVTLT-NIR. Optimization of elution conditions included elution time (10 or 30 min) or addition of acetonitrile in the elution solvent. Blank sample (A), elution with 90/10 H_2O/ACN containing 0.1% TFA, in 10 min (B), elution with 90/10 H_2O/ACN containing 0.1% TFA, in 30 min (C), elution with 50/50 H_2O/ACN containing 0.1% TFA, in 10 min (D). Final condition: 30 min elution in 90/10 H_2O/ACN containing 0.1%TFA.

```
SP|P11140|ABRA_ABRPR ---------------------------------QDRPIKFSTEGATSQSYKQFIEALRE 26
SP|P11140|ABRA_ABRPR RLRGGLIHDIPVLPDPTTLQERNRYITVELSNSDTESIEVGIDVTNAYVVAYRAGTQSYF 86
SP|P11140|ABRA_ABRPR LRDAPSSASDYLFTGTDQHSLPFYGTYGDLERWAHQSRQQIPLGLQALTHGISFFRSGGN 146
SP|P11140|ABRA_ABRPR DNEEKARTLIVIIQMVAEAARFRYISNRVRVSIQTGTAFQPDAAMISLENNWDNLSRGVQ 206
SP|P11140|ABRA_ABRPR ESVQDTFPNQVTLTNIRNEPVIVDSLSHPTVAVLALMLFVCNPPNANQSPLLIRSIVEKS 266
SP|P11140|ABRA_ABRPR KICSSRYEPTVRIGGRDGMCVDVYDNGYHNGNRIIMWKCKDRLEENQLWTLKSDKTIRSN 326
SP|P11140|ABRA_ABRPR GKCLTTYGYAPGSYVMIYDCTSAVAEATYWEIWDNGTIINPKSALVLSAESSSMGGTLTV 386
SP|P11140|ABRA_ABRPR QTNEYLMRQGWRTGNNTSPFVTSISGYSDLCMQAQGSNVWMADCDSNKKEQQWALYTDGS 446
SP|P11140|ABRA_ABRPR IRSVQNTNNCLTSKDHKQGSTILLMGCSNGWASQRWVFKNDGSIYSLYDDMVMDVKGSDP 506
SP|P11140|ABRA_ABRPR SLKQIILWPYTGKPNQIWLTLF 528
```

Figure S3. Sequence coverage of abrin at 250 ng/mL by MALDI-TOF. Abrin peptides identified by MALDI-TOF MS are highlighted in green.
